# Association between Intake of Fermented Dairy Product and Diet Quality, Health Beliefs in a Representative Sample of Polish Population

**DOI:** 10.3390/nu14235018

**Published:** 2022-11-25

**Authors:** Anna Danielewicz, Jakub Morze, Katarzyna Staniewska, Aneta Dąbrowska, Tomasz Sawicki, Zhennai Yang, Maria Baranowska, Małgorzata Darewicz, Justyna Żulewska, Bogusław Staniewski, Katarzyna E. Przybyłowicz

**Affiliations:** 1 Department of Human Nutrition, University of Warmia and Mazury in Olsztyn, 10-718 Olsztyn, Poland; 2 Department of Cardiology and Internal Diseases, University of Warmia and Mazury in Olsztyn, 10-082 Olsztyn, Poland; 3 Department of Commodity Science and Food Analysis, University of Warmia and Mazury in Olsztyn, 10-726 Olsztyn, Poland; 4 Department of Dairy Science and Quality Management, University of Warmia and Mazury in Olsztyn, 10-719 Olsztyn, Poland; 5 Beijing Advanced Innovation Center for Food Nutrition and Human Health, Beijing Engineering and Technology Research Center of Food Additives, Beijing Technology and Business University, Beijing 100048, China; 6 Deparment of Food Biochemistry, University of Warmia and Mazury in Olsztyn, 10-726 Olsztyn, Poland

**Keywords:** dairy, fermented dairy, Mediterranean diet, diet quality, health benefits, health beliefs, health concern

## Abstract

This study aimed to evaluate the association of diet quality and perception of consumption benefits with intake of fermented dairy products in a representative sample of the Polish population. The study was carried out in February 2020 and involved 2009 men and women randomly sampled from the representative Polish population stratified into two age groups (19–30 and 66–75 years). Dairy product intake was evaluated using a qualitative food frequency questionnaire. Diet quality was assessed by calculating the Mediterranean Diet Adherence Screener (MEDAS) score. The perceived health benefit of dairy product consumption was assessed by a literature-based questionnaire. The Health Concern Scale was used to measure participants’ attitudes toward health. The median intake of fermented dairy products was 0.8 portion/day (IQR: 0.4–1.6). Intake of fermented dairy products was associated with a higher MEDAS score. We observed that people with the highest intake of fermented dairy products consumed more oils, vegetables, wine, legumes, fish and seafood, sweets and pastries, nuts, had a higher preference for white meat and were more likely to report their perceived benefits to maintain body weight, reduce cardiovascular risk, and improve immune and dental health. Moreover, a high intake of fermented dairy products was positively related to paying more attention to health. Our study identified patterns of health behaviors associated with the frequent consumption of fermented dairy products. We observed that the intake of fermented dairy products is associated with better diet quality, consumer self-consciousness, and a greater attitude toward personal health.

## 1. Introduction

Unhealthy diets contribute to the increasing burden of non-communicable diseases.

Annually, noncommunicable diseases (NCDs) kill 41 million people each year and 17 million people die from them before age 70. Cardiovascular diseases account for most NCDs with 17.9 million deaths, followed by cancers (9.3 million), chronic respiratory diseases (4.1 million), and diabetes (2.0 million including kidney disease deaths caused by diabetes) [1]. Consumption of dairy products has the potential to protect against NCDs [2]. More research suggests that supplementation with dairy products and healthy eating patterns, such as the Mediterranean diet, may provide a numerous health benefits and improve health outcomes [3].

The most common examples of fermented dairy products are fermented milk items (e.g., yogurt, cultured cream, buttermilk, and kefir, although many variations of these products exist based on historical practices, geography, and type of milk), butter, and cheeses [2]. Fermented dairy products have existed for millennia as an outstanding way to preserve milk, and to provide safe food with desirable sensorial properties and numerous health benefits [4,5]. It should also be emphasized that dairy products constitute a heterogeneous food group, consisting of a large number of products that differ in both the nutritional composition, such as fat content, and the structure of the dairy matrix, which indicates that certain fermented milk products may have different effects on blood lipid levels [6]. Moreover, the presence of bacteria, fungi and yeast, through their metabolism and proteolytic action, can promote the release of fatty acids and peptides as well as minerals and vitamins (such as vitamins A, B_1_, B_2_, B_6_, B_12_, niacin, pantothenic acid and folic acid, as well as vitamin D, calcium, phosphorus, potassium, magnesium, zinc and potassium iodide [7]). Thereby, increasing their bioavailability from milk products may result in additional benefits for the body [8]. These effects may include the modulation of immune function, healthy intestinal microbiota, elimination of pathogens, decreased allergy, and other benefits that may slow down the deterioration of health [9]. An important issue that should also be emphasized is that of milk fat. After years of controversy, with many guidelines recommending a reduced intake of full-fat dairy products, in preference for the consumption of low or nonfat dairy foods, current knowledge points to the more appropriate recommendation of moderate consumption of full-fat dairy foods as part of a healthy lifestyle [10]. Saturated fats (SFA), cholesterol, and the caloric content of dairy products, have been the basis of past arguments against dairy fats. SFA has been considered to affect cardiovascular health negatively, but nowadays, a current meta-analysis of cohort studies suggested that total fat, SFA, MUFA, and PUFA intakes were not associated with an increased risk of cardio-vascular disease [10,11].

The presence of bacteria in fermented dairy products is very variable and depends mainly on the storage time. The shorter the time, the higher the values observed and technological process used. The main species in fermented milk products are *L. lactis*, *S. salivarius* ssp. *thermophilus* (*S. thermophilus)* and *lactobacilli* such as *L. delbrueckii* ssp in yoghurts and fermented milk and *L. helveticus* in Swiss cheeses [5]. It should be emphasized here that particular interest in the consumption of fermented dairy foods can be credited to the explosion of research into the human microbiome [12,13]. Studies have demonstrated that diet influences the structure and function of the gut microbiota [14,15]. It is believed that the consumption of fermented foods containing probiotics—“live microorganisms which, when administered in adequate amounts, confer a health benefit on the host” [16,17]—is an effective way to introduce potentially beneficial microorganisms to the intestinal tract and help manage a wide range of disorders. Those among others include disorders associated with gut microbial dysbiosis, such as behavior and (gut-)brain disorders, inflammatory bowel disease, irritable bowel syndrome, and coeliac disease, as well as extra-intestinal disorders, including allergy, asthma, obesity, metabolic syndrome, cardiovascular disease and cancer [17,18,19,20]. Another critical aspect affecting the health impact of fermented foods is the number of live microorganisms provided by the consumption of fermented foods. In a review by Rezac et al. [21], many fermented foods (cheese, yoghurt, sausages, vegetables, cereals, sour beer, kombucha, fermented fish, and tempeh) were found to contain 10^5^–10^7^ colony forming units (CFU) of LAB/(mL or g), with cultured dairy products containing up to 10^9^ CFU/(mL or g) [17].

Thus, it seems necessary to increase the scale of educational activities related to the promotion of the consumption of fermented milk products to reduce the occurrence of noncommunicable diseases. The intention to maintain health may be one of the reasons for choosing particular groups of foods. The high share of fermented dairy products in Polish diet results from Poles’ belief in the benefits of their consumption [22]. However, these beliefs only sometimes concern the perception of all possible beneficial health effects. Thus, this study aimed to evaluate the association between diet quality, perception of consumption benefits, and fermented dairy product intake in a representative sample of the Polish population.

## 2. Materials and Methods

### 2.1. Sample Collection

The attempt to survey Poles’ opinions on dairy products and their aspect of innovation was representative, nationwide and included 2009 Poles aged 19–30 and 66–75. The survey was conducted using computer assisted personal interviews (CAPI) in January and February 2020. The sample for the survey had a quota-random character. The population characteristics that were taken into account in compiling the sample were: voivodship, borough size class, gender and age of the respondent in 2 categories: 19–30 years old and 66–75 years old. Due to the lack of data on food consumption, the studied sample consisted of1695 people.

### 2.2. Questionnaires

The structured questionnaire concerned the study of consumer behavior on the market of innovative dairy products with pro-healthy properties and the study of expectations towards these products. The questionnaire covered 8 thematic groups and contained 83 questions giving 302 result variables.

Dairy product consumption was assessed using a qualitative food frequency questionnaire where questions were created based on the validated KomPAN questionnaire [23] and Codex Alimentarius dairy food grouping [24]. Total fermented dairy product consumption was calculated as a sum of natural and sweetened fermented dairy products and rennet cheeses.

Diet quality was described with the German version of the Mediterranean Diet Adherence Screener (MEDAS) score [25]. The questionnaire consists of 14 questions concerning the consumption of selected food groups. However, an original question relating to a dish with a traditional sauce of tomatoes, garlic, onion, or leeks sautéed in olive oil, was changed to servings of whole-grain cereal products. The questionnaire result allows for assessing to what extent the diet of the examined person is consistent with the Mediterranean diet pattern. This allowed for selecting groups whose diet was consistent with the principles of proper nutrition or was different from theirs.

The Health Concern Scale (HCS) allowed to assess the respondent’s attitudes to health. The questionnaire consists of 10 statements concerning the interest in health and the relationship between excessive consumption of sugar, fat, salt, cholesterol, and food additives and selected diseases [26].

The questions related to the health benefits of regular consumption of all analyzed dairy product was created based on the literature. The questions concerned weight management, heart, bone, digestive and dental health, and immune defense; however, respondents could also indicate no benefits.

### 2.3. Confounding Factors

Participants declared their weight, height and waist circumference. On this basis body mass index (BMI) was calculated (kg/m^2^) and classified as underweight (<18.50 kg/m^2^) normal (18.50–24.99 kg/m^2^), or overweight and obesity (≥25.00 kg/m^2^). Other confounders which were taken into account were: age cohort (19–30 y, 66–75 y), gender (women, men), smoking status (current, former, never), sleeping time (<6 h, 6–9 h, >9 h), TV watching time (<2 h, 2–4 h, 4–6 h, 6–8 h, 8–10 h, >10 h), education level (primary, secondary, vocational, higher, not wish to answer), economic status (very poor, poor, medium, good, very good, not wish to answer).

### 2.4. Statistical Analysis

Variables were presented as a median and interquartile range (IQR) if were continuous and as a percentage and number if were categorical. Differences of continuous variables across quartiles of total fermented dairy product consumption were evaluated using Kruskal-Wallis test and Dunn’s post-hoc test. Differences of categorical variables were obtained using chi-square or Fisher’s exact test. The associations between quintiles of total fermented dairy products consumption and MEDAS, its component, HCS and health benefits were evaluated by the generalized linear model. In all analysis, covariate adjustment set included age cohort, gender, smoking status, sleeping time, TV watching time, education level, economic status (all categorical). Due to skewed distributions input data were first log-transformed and analyzed with the generalized linear models. For clarity of presentation results were presented as back-transformed (exponentiated) least square means and their 95% confidence interval (95% CI). In similar manner, associations of HCS, perceived health benefits and MEDAS with consumption of fermented dairy products were evaluated using general linear model using the same adjustment set, as indicated above.

The significance level was set to *p* < 0.05. The statistical analysis was carried out using STATISTICA software (version 13.1 PL; StatSoft Inc.: Tulsa, OK, USA/Statsoft Inc.: Kraków, Poland).

## 3. Results

Table 1 shows the basic characteristics of the studied sample by quintiles of total fermented dairy products. The median consumption frequency was 0.8 (0.4; 1.6) portions/day. Participants in Q5 compared with Q3 declared higher waist circumference (87 cm vs. 80 cm, respectively) and in the age cohort of 19–30 years, were younger (25 years vs. 27 years, respectively). Participants who consumed fermented dairy products more frequently were more likely to be women, watch TV more than 6 h per day and less likely to sleep 6–9 h.

Table 2 presents the association of MEDAS score and its components with total dairy fermented products consumption. Unadjusted data were presented in Table S1. Frequency of dairy fermented products consumption was associated with higher MEDAS score (Q1 = 5.6 IQR: 5.3; 5.9 to Q5 = 6.2 IQR: 5.9; 6.6, *p_trend_* < 0.001). We observed that the frequency of dairy fermented products consumption was positively associated with frequent consumption of oils, vegetables, wine, legumes, fish and seafood, sweets and pastries, nuts (for all *p_trend_* < 0.001) and a higher preference for white meat (*p_trend_* = 0.014).

The results of the association between the total frequency of dairy fermented products consumptions and HSC, perceived health benefits are shown in Table 3. A high intake of fermented dairy products was positively related to paying more attention to health. Participants were more likely to report perceived health benefits to maintain normal body weight (Q5 = 39.6% vs. Q1 = 26.3%), reducing cardiovascular risk (Q5 = 52.3% vs. Q1 = 26.6%), and improving immune (Q5 = 67.3% vs. Q1 = 50.3%) and dental health (Q5 = 36.9% vs. Q1 = 27.3%) together with higher consumption of dairy fermented products. Participants who did not indicate benefits for health were negatively associated with fermented dairy product consumption (Q5 = 20.5% vs. Q1 = 51.6%).

Table 4 shows association between health beliefs attributed to the consumption of subgroups of fermented dairy products and the intake of respective subgroups. Heart health was the only belief associated with the intake of naturally fermented dairy products. All health beliefs, except improved dental health for flavored products and improved bone health, were associated with more frequent intake of those product subgroups. Regular consumers of all fermented dairy product subgroups were less likely to indicate no benefit from their consumption (b from −0.176 to −0.096) Higher health awareness (HCS) and better diet quality were associated with higher intake of natural (b = 0.037 and b = 0.005) and flavored (b = 0.027 and b = 0.006) dairy products, but not rennet cheese. Unadjusted data were presented in Table S2.

## 4. Discussion

This study investigated dietary, lifestyle, and behavioral determinants of fermented dairy product intake in a representative sample of the Polish population. We observed that people with higher diet quality, as assessed by adherence to the Mediterranean diet. With respect to health attitudes, regular consumers paid more attention to their health. Moreover, a higher intake of fermented dairy products was more frequently motivated by perceived benefits for musculoskeletal, circulatory, digestive, and immunological systems. However, these motivations were mainly attributed to flavored fermented products and rennet cheeses. Similar results were obtained by Sajdakowska [23] when examining the importance of health aspects in Polish consumer choices of dairy product in the population of respondents from 16 voivodships in Poland who were aged 21 and over. In this study regarding opinions on the perception of dairy products, respondents declared the highest level of agreement with the statement that dairy products are a rich source of protein and that dairy products, particularly yoghurts, kefirs and butter milk, have a beneficial influence on immunity [23].

When consumed in enough proportions as part of a balanced diet on a regular basis, fermented dairy foods provide health benefits. In addition, these functional foods also provide the body with critical nutrients [3]. Fermented dairy products are a primary source of prebiotics and probiotics. Furthermore, those products offer a variety of compounds, including proteins, peptides, oligosaccharides, vitamins and organic acids (including fatty acids) with distinct health effects [9]. An important issue related to the consumption of fermented milk products is that the consumer should also be aware that fermentation of milk into cheese and yogurt has been shown to increase the levels of free amino acids detected in plasma, including α-amino butyric acid, alanine, asparagine, cysteine, glycine, glutamine, histidine, isoleucine, leucine, lysine, methionine, ornithine, phenylalanine, proline, serine, threonine, tryptophan, tyrosine, and valine [27,28,29]. However, it should be taken into consideration that, while a moderate presence of free amino acids in the diet may have a beneficial effect on skeletal muscle synthesis and the fight against microbial infections [30,31], excessive concentrations can result in increased inflammation caused by elevated intestinal permeability, increased risk of cell atherosclerosis, impair the immune response to infection, and cause allergic reactions [31,32]. Increased levels of free fatty acids may be beneficial for health due to promoting growth of the host intestinal microbiota, enhancing immunity, regulating blood pressure, increasing the anabolism of muscle proteins, or inhibiting the binding of pathogens [27,33]. Very important as recent studies indicate about milk fat that higher intake of short-chain saturated fatty acids (SCSFAs) and medium-chain saturated fatty acids (MCSFAs) was inversely associated with dyslipidemia and diabetes (OR = 0.43, 95% CI: 0.23, 0.82 and OR = 0.25, 95% CI: 0.09, 0.72, respectively). Among MUFAs, C18:1 was inversely associated with hypertension and diabetes fatty acid [34]. Moreover, many micronutrients (calcium, phosphorus, A and B vitamins, potassium, zinc, and choline) also have a higher bioavailability in yoghurt than in raw milk due to the acidity and fermentation process [6].

The benefits of fermented dairy product intake can be explained by immunological, anti-carcinogenic, immunomodulatory, antiallergic, antioxidative, as well as lipid-, glucose-, and blood-pressure-lowering effects [20]. Considering a high intake of fermented products as a part of healthy, sustainable dietary pattern shows the potential to expand its health effects. A number of experimental studies [3,35,36,37] support this point, showing that healthy diets supplemented with fermented dairy products might be superior to diets that exclude those products. Although guidelines are lacking for the minimum dose of live microorganisms that should be consumed, the European Union (EU) health claim for yoghurt and “improved lactose tolerance” stipulates that at least 10^8^ CFU of live starter microorganisms per gram of yogurt [29,38]. In the recent research work of Derrien et al. [39] predicted that ingesting a dose of 10^10^ ingested bacterial cells would be sufficient to drastically shift the composition of the gut microbiota and impact the immune and neuroendocrine responses of the host.

In particular, the promotion of fermented dairy product intake to improve overall diet quality should be considered in countries and societies where those products are available at a relatively low cost [22,40]. As discussed above, there is a growing popular consensus that the consumption of fermented foods results in positive health effects. Much of this is driven by popular observations that generally fermented foods use unprocessed raw ingredients, contain little or no added preservatives, colors or flavorings, and are made using long-established, sustainable, and in many cases traditional technologies [41]. Consumers may be attracted to the concept that these are “live foods” containing natural and diverse microbiota [42].

The second significant implication reflects the target group promoting fermented dairy product intake. Our findings indicated that lower intake is driven by young age and male sex. The burden of health risks and exposures in a group of young adults is a key determinant of their status in further life. In this context, males are exposed more significant health harms by higher smoking rates, excessive alcohol consumption, or lower diet quality. Based on our findings and health benefits based on published literature, both groups can be considered as targets for interventions aiming to increase the intake of fermented dairy products. The authors of this work have a particular awareness that the consumption of dairy, particularly low-fat dairy, is advocated in most dietary guidelines worldwide. Still, there is, therefore, a necessity to expand such research could eventually have an impact on public health, such a refining guidelines, especially for fermented dairy foods, and a better understanding of the composition of different fermented food products as part of healthy, sustainable diets [17,29]. This approach can help convince consumers that consuming fermented dairy products helps maintain health and prevent disease ispart of an effort to increase life expectancy and improve life quality. To establish long-term, positive relations between the police maker and the final consumer, creating a coherent, transparent, well-thought-out communication strategy with the consumer is necessary. It is also important that the information communicated should be logical, uncomplicated and consistent, and communicated using the largest possible number of communication channels to increase the consumption of fermented milk products in various age groups adapted to their health expectations and health beliefs predictive of dieting intentions and behavior. Providing consumers with reliable scientific knowledge about the possible impact of food products on health, aimed at various social groups, will enable them to form the basis of new favorable nutritional beliefs, which translate into the inclusion of these products in the diet. Research that addresses these critical issues and explores beliefs about the benefits of consuming fermented dairy products in the broader context in which consumption decisions are made can be used to best target nutritional prevention efforts regarding social health. 

Despite the strengths of the survey, such as sample size, nationally representative, randomly selected study sample and extensive covariate adjustment, its limitations cannot be neglected. Firstly, this was a cross-sectional study, which makes it impossible to identify cause-and-effect relationships. In addition, the age groups chosen for the study may be a limitation. They make it impossible to assess the relationship between changing eating habits over a lifespan. However, such a selection of respondents resulted from the original assumptions of the project. Another limitation are the use of self-reported questionnaires and declared values of anthropometric characteristics. Despite the use of validated tools, they rely on respondents’ memory bias and are prone to overestimating or underestimating the frequency of food consumption. 

## 5. Conclusions

In conclusion, our study identified patterns of health behaviors associated with frequent consumption of fermented products. We observed that the intake of fermented dairy products is associated with better diet quality, consumer self-consciousness, and a greater attitude to own health. Therefore, it seems necessary to develop pro-health attitudes and beliefs that encourage consumption of these products within the framework of national nutritional guidelines, which may protect and help and contribute to reducing the incidence of chronic non-communicable diseases in various age groups of the population.

## Figures and Tables

**Table 1:** Basic characteristics.

	Total Fermented Dairy Products Consumption					*p*
	Q1	Q2	Q3	Q4	Q5	
Fermented dairy product consumption, times/d †	0.2 (0.1; 0.2)	0.4 (0.3; 0.7)	0.8 (0.7; 0.9)	1.2 (1.2; 1.3)	2.1 (1.7; 2.6)	
Age cohort 19–30 y, years †	26 (23; 28)	26 (23; 29)	27 (24; 29) ^a^	26 (22; 28)	25 (21; 28) ^a^	0.022
Age cohort 66–75 y, years †	69 (67; 71)	69 (67; 71)	68 (67; 70)	69 (67; 71)	69 (67; 71)	0.310
Gender (women)	48.1 (148)	51.3 (180)	58.7 (186)	53.2 (166)	59.0 (240)	0.015
Waist circumference, cm †	86 (75; 100)	83 (70; 95)	80 (70; 90) ^a^	85 (75; 96)	87 (76; 98) ^a^	0.016
Body mass index-men, kg/m^2^ †	25.9 (24.0; 28.7)	26.0 (22.9; 28.0)	25.6 (23.6; 27.8)	26.0 (23.7; 28.7)	26.0 (23.4; 28.7)	0.613
Body mass index-women, kg/m^2^ †	24.5 (20.7; 29.3)	23.4 (21.4; 27.7)	23.8 (21.3; 26.7)	24.2 (22.0; 27.6)	24.2 (21.7; 27.8)	0.520
% Overweight and obese	53.7 (123)	51.5 (138)	48.4 (119)	53.3 (119)	50.9 (172)	0.773
Smoking status (current)	20.1 (62)	24.8 (87)	24.3 (77)	23.7 (74)	27.3 (111)	0.290
Physical activity (MVPA)	61.0 (188)	65.8 (231)	68.8 (218)	65.4 (204)	61.2 (249)	0.170
TV watching time (more than 6 h)	12.3 (38)	17.4 (61)	16.4 (52)	18.9 (59)	24.3 (99)	0.001
Sleeping time (6–9 h)	79.2 (244)	83.2 (292)	77.9 (247)	76.0 (237)	74.0 (301)	0.034
Economic status (high and very high)	25.0 (77)	28.8 (101)	25.9 (82)	24.4 (76)	28.0 (114)	0.643
Education level (higher)	13.6 (42)	15.1 (53)	18.3 (58)	11.9 (37)	13.0 (53)	0.167

Data are presented as % and number or (where †) median and interquartile range (IQR). The sample size may vary slightly in each variable due to missing data. MVPA, moderate-to-vigorous physical activity. *p* value < 0.05: significance for Kruskal-Wallis test (Dunn’s post-hoc test—superscript letter e.g., ^a^—denotes pair with a significant between-group effect) or Pearson’s chi square test.

**Table 2:** Association between MEDAS, its components and total fermented dairy products consumption.

	Total Fermented Dairy Products Consumption					*p*_trend_
	Q1	Q2	Q3	Q4	Q5	
MEDAS	5.6 (5.3; 5.9)	5.8 (5.4; 6.1)	5.7 (5.4; 6.1)	6.0 (5.6; 6.3)	6.2 (5.9; 6.6)	<0.001
Plant oils as main (yes) †	73.1 (225)	83.5 (293)	86.4 (274)	87.2 (272)	83.1 (338)	0.096
Plant oils, times/d	1.7 (1.4; 2.0)	1.7 (1.5; 2.0)	2.1 (1.8; 2.4)	2.0 (1.7; 2.3)	2.3 (2; 2.6)	<0.001
Vegetables, times/d	2.7 (2.3; 3.0)	2.6 (2.3; 3.0)	2.5 (2.2; 2.8)	2.6 (2.3; 3.0)	3.1 (2.8; 3.5)	<0.001
Fruits and juices, times/d	4.7 (4.2; 5.3)	5.2 (4.6; 5.8)	5.7 (5.1; 6.3)	5.5 (4.9; 6.1)	5.1 (4.6; 5.7)	0.451
Red meat, times/d	4.8 (4.3; 5.5)	5.1 (4.5; 5.9)	5.2 (4.6; 5.9)	6.0 (5.3; 6.8)	5.6 (5.0; 6.3)	0.168
Butter and cream, times/d	2.6 (2.2; 2.9)	2.5 (2.2; 2.9)	3.1 (2.7; 3.5)	2.6 (2.3; 3.0)	2.9 (2.6; 3.3)	0.084
Sweetened beverages, times/d	4.0 (3.3; 4.8)	5.0 (4.1; 6.0)	4.9 (4.1; 5.8)	4.6 (3.8; 5.6)	4.5 (3.8; 5.4)	0.687
Wine, times/wk	1.4 (1.1; 1.8)	1.6 (1.2; 2.1)	1.4 (1.1; 1.9)	1.9 (1.4; 2.4)	2.5 (2.1; 3.1)	<0.001
Legumes, times/wk	1.2 (1.0; 1.5)	1.4 (1.2; 1.7)	1.7 (1.5; 2.1)	1.7 (1.4; 2.0)	2.0 (1.7; 2.4)	<0.001
Fish and seafood, times/wk	1.3 (1.1; 1.6)	1.6 (1.4; 1.9)	1.7 (1.4; 2.0)	1.9 (1.6; 2.2)	2.1 (1.9; 2.4)	<0.001
Sweets and pastries, times/wk	3.7 (3.2; 4.2)	4.0 (3.5; 4.6)	4.5 (4.0; 5.1)	4.4 (3.8; 5.0)	4.9 (4.4; 5.5)	<0.001
Nuts, times/wk	0.6 (0.4; 0.9)	0.8 (0.5; 1)	1.1 (0.8; 1.4)	1.1 (0.8; 1.5)	1.6 (1.3; 2.1)	<0.001
Preferable white meat (yes) †	71.4 (220)	75.2 (264)	73.2 (232)	79.8 (249)	80.6 (328)	0.014
Whole grain products, times/d	2.5 (2.1; 2.9)	2.5 (2.1; 2.9)	2.6 (2.3; 3.1)	2.7 (2.4; 3.2)	2.6 (2.3; 3.0)	0.179

Data are presented as back-transformed least square means and 95% confidence interval (CI) or (where †) as % and number. The sample size may vary slightly in each variable due to missing data. MEDAS, MEDAS, Mediterranean Diet Adherence Screener. All continuous data were adjusted for age cohort, gender, smoking status, sleeping time, TV watching time, education level, economic status (all categorical). *p* value < 0.05: significance for Wald’s test.

**Table 3:** Association of health concern scale and health beliefs with total fermented dairy products consumption.

	Total Fermented Dairy Products Consumption					*p*_trend_
	Q1	Q2	Q3	Q4	Q5	
**% (n)**	18.2 (308)	20.7 (351)	18.7 (317)	18.4 (312)	24.0 (407)	
HCS †	37.1 (35.3; 39.0)	36.6 (34.8; 38.5)	36.2 (34.4; 38.0)	37.3 (35.5; 39.2)	39.5 (37.7; 41.4)	0.002
Normal body weight	26.3 (81)	29.3 (103)	38.2 (121)	38.8 (121)	39.6 (161)	0.001
Healthy heart	26.6 (82)	32.5 (114)	39.4 (125)	46.2 (144)	52.3 (213)	<0.001
Healthy bones	56.5 (174)	64.7 (227)	71.9 (228)	71.2 (222)	67.6 (275)	0.075
Improved immunity	50.3 (155)	61.3 (215)	67.2 (213)	68.6 (214)	67.3 (274)	0.002
Healthy digestive track	59.1 (182)	63.8 (224)	68.5 (217)	70.8 (221)	61.2 (249)	0.449
Healthy teeth	27.3 (84)	29.3 (103)	37.5 (119)	36.5 (114)	36.9 (150)	<0.001
Not bring benefits	51.6 (195)	37.3 (131)	26.2 (83)	29.2 (91)	20.6 (84)	<0.001

Data are presented as % and number or (where †) median and interquartile range (IQR). HCS, health concern scale. All continuous data were adjusted for age cohort, gender, smoking status, sleeping time, TV watching time, education level, economic status (all categorical). *p* value < 0.05: significance for Wald’s test.

**Table 4:** Association of health concern scale, health beliefs and diet quality with consumption of fermented products according to their subgroups.

	Natural Fermented Dairy Products	Flavored Fermented Dairy Products	Rennet Cheese
HCS	**0.037 (0.005)**	**0.027 (0.005)**	−0.002 (0.006)
Normal body weight	0.038 (0.022)	**0.084 (0.025)**	**0.15 (0.029)**
Heart health	**0.066 (0.021)**	**0.154 (0.022)**	**0.067 (0.027)**
Bone health	−0.017 (0.018)	**0.044 (0.018)**	0.042 (0.021)
Immune defence	0.024 (0.019)	**0.039 (0.019)**	**0.07 (0.023)**
Digestive health	−0.023 (0.018)	**0.021 (0.019)**	**0.077 (0.023)**
Dental health	0.008 (0.022)	0.055 (0.024)	**0.118 (0.027)**
No benefits	**−0.096 (0.037)**	**−0.176 (0.024)**	**−0.12 (0.025)**
MEDAS	**0.005 (0.001)**	**0.006 (0.001)**	0.002 (0.001)

Data are presented as unstandardized regression coefficient (standard error) from a model adjusted for age cohort, gender, smoking status, sleeping time, TV watching time, education level, economic status (all categorical). HSC, health concern scale. MEDAS, Mediterranean Diet Adherence Screener. Bolded are coefficients at *p* < 0.05.

## Data Availability

Due to ethical restrictions and participant confidentiality, data cannot be made publicly available. However, data from the DairyFunInn study are available upon request, for researchers who meet the criteria for access to confidential data. Data requests can be sent to the DairyFunInn study coordinator (Katarzyna Eufemia Przybyłowicz).

## Supplementary Materials

The following supporting information can be downloaded at: https://www.mdpi.com/article/10.3390/nu14235018/s1, Table S1: Association between MEDAS, its components and total fermented dairy products consumption; Table S2: Association of health concern scale, health beliefs and diet quality with consumption of fermented products according to their subgroups
