# Supplementary material for: Association between Intake of Fermented Dairy Product and Diet Quality, Health Beliefs in a Representative Sample of Polish Population
Source: Nutrients. 2022 Nov 25;14(23):5018. doi: 10.3390/nu14235018 (PMC9736614; doi:10.3390/nu14235018)
Supplement: Supplementary file 1 [file nutrients-14-05018-s001.zip › nutrients-2004700-supplementary.pdf]

Supplementary materials

Association between intake of fermented dairy product and diet quality, health beliefs in a representative sample of Polish population

Anna Danielewicz[1*], Jakub Morze[1,2], Katarzyna Staniewska[3], Aneta Dąbrowska[4], Zhennai Yang[5], Maria Baranowska[4], Małgorzata Darewicz[6], Justyna Żulewska[4], Bogusław Staniewski[4], Katarzyna E. Przybyłowicz[1*,]

1 Department of Human Nutrition, University of Warmia and Mazury in Olsztyn, Polan
2 Department of Cardiology and Internal Diseases, University of Warmia and Mazury in Olsztyn, Poland
3 Department of Commodity Science and Food Analysis, University of Warmia and Mazury in Olsztyn, Poland
4 Department of Dairy Science and Quality Management, University of Warmia and Mazury in Olsztyn, Poland
5 Beijing Advanced Innovation Center for Food Nutrition and Human Health, Beijing Engineering and Technology Research Center of Food Additives, Beijing Technology and Business University, Beijing, P.R. China 100048
6 Chair of Food Biochemistry, University of Warmia and Mazury in Olsztyn, Poland

Table S1. Association between MEDAS, its components and total fermented dairy products consumption.

	Total fermented dairy products consumption					P_{trend}
	**Q1**	**Q2**	**Q3**	**Q4**	**Q5**	
MEDAS	6 (4; 7)	6 (5; 7)	6 (5; 7)	6 (5; 7)	6 (5; 8)	<0.001
Plant oils as main (yes)†	73.1 (225)	83.5 (293)	86.4 (274)	87.18 (272)	83.1 (338)	<0.001
Plant oils, times/d	1 (1; 2)	1 (1; 2)	1 (1; 3)	2 (1; 2)	2 (1; 3)	<0.001
Vegetables, times/d	2 (1; 3)	2 (1; 3)	2 (1; 2.5)	2 (1; 3)	2 (2; 4)	<0.001
Fruits and juices, times/d	4 (2; 7)	5 (3; 7)	6 (3; 8)	5 (3; 7)	5 (3; 7)	0.075
Red meat, times/d	3 (2; 5)	3 (2; 4)	3 (2; 5)	3 (2; 5)	3 (2; 5)	<0.001
Butter and cream, times/d	2 (1; 3)	2 (1; 3)	2 (1; 3)	2 (2; 3)	2 (2; 3)	0.003
Sweetened beverages, times/d	2 (0; 4)	2.5 (0; 5)	3 (1; 5.5)	2 (0; 4)	3 (1; 5)	<0.001
Wine, times/wk	0 (0; 0.5)	0 (0; 1)	0 (0; 1)	0 (0; 1)	1 (0; 3)	<0.001
Legumes, times/wk	1 (0; 1)	1 (0; 2)	1 (0; 2)	1 (1; 2)	2 (1; 3)	<0.001
Fish and seafood, times/wk	1 (0; 1)	1 (1; 2)	1 (1; 2)	1 (1; 2)	1 (1; 3)	<0.001
Sweets and pastries, times/wk	3 (2; 4)	3 (2; 5)	3 (2; 5)	3 (2; 5)	4 (2; 6)	<0.001
Nuts, times/wk	0 (0; 1)	0 (0; 1)	0 (0; 2)	1 (0; 2)	1 (0; 2)	<0.001
Preferable white meat (yes)†	71.4 (220)	75.2 (264)	73.2 (232)	79.8 (249)	80.6 (328)	0.015
Whole grain products, times/d	2 (1; 3)	2 (1; 3)	2 (1; 3)	2 (1; 4)	2 (1.5; 3)	0.019

Data are presented as median and interquartile range (IQR) or (where†) as % and number. The sample size may vary slightly in each variable due to missing data. MEDAS, MEDAS, Mediterranean Diet Adherence Screener. ~~p~~ P value <0.05: significance for Kruskal-Wallis test (Dunn's post-hoc test) or Pearson's chi square test.

Table S2. Association of health concern scale, health beliefs and diet quality with consumption of fermented products according to their subgroups.

	**Natural fermented dairy products**	**Flavoured fermented dairy products**	**Rennet cheese**
HCS	**0.158676**	**0.122644**	0.042318
Normal body weight	**0.086472**	**0.083768**	**0.049415**
Heart health	**0.169006**	**0.188995**	**0.106801**
Bone health	**0.088887**	**0.053926**	**0.054818**
Immune defence	**0.124786**	**0.134883**	0.037514
Digestive health	0.031016	0.003205	0.018248
Dental health	**0.048255**	0.035988	0.045201
No benefits	**-0.187648**	**-0.235776**	**-0.154978**
MEDAS	**0.169951**	**0.133693**	0.016360

Data are presented as regression coefficient. HSC, health concern scale. N=1695. MEDAS, Mediterranean Diet Adherence Screener. Bolded are coefficients at $p<0.05$ (Pearson's test)
